# Are insect bites responsible for the rise in summer flucloxacillin prescribing in United Kingdom general practices?

**DOI:** 10.1093/fampra/cmad051

**Published:** 2023-05-06

**Authors:** Jane Wilcock, Kamila Hawthorne, Joanne Reeve, Clare Etherington, Katharine Alsop, Joanna Bircher, Douglas McKechnie, Stephen Granier, Daniel Newport, Simon Wright, James Larcombe, Chinonso Ndukauba, Nitharnie Anastasius

**Affiliations:** GP Silverdale Medical Practice, Silverdale Medical Practice, Salford, United Kingdom; Professor, Academic Office—312 Second Floor Grove Building Singleton Campus, Swansea University Medical School, Wales, United Kingdom; GP, Meddygfa Glan Cynon, Cynon Vale Medical Practice, Ty Calon Lan, Oxford Street, Mountain Ash, Wales, United Kingdom; Professor, Hull York Medical School, Academy of Primary Care, United Kingdom; GP, Ridgeway Surgery, Harrow, London, United Kingdom; GP, Nightingale Valley Practice, Brislington, Bristol, United Kingdom; GP, Lockside Medical Centre, Stalybridge, Tameside, United Kingdom; GP, University College London, Research Department of Primary Care and Population Health; Holborn Medical Centre, London, United Kingdom; GP, Whiteladies Medical Group, Whatley Road, Clifton, Bristol, United Kingdom; Medicine Trainee, University Hospitals Birmingham NHS Foundation Trust, Birmingham, United Kingdom; GP, Walkden Medical Practice, Salford, United Kingdom; GP, Sedgefield, Co Durham, United Kingdom; GP, Whiteladies Medical Group, Whiteladies, Whatley Road, Clifton, Bristol, United Kingdom; GP Ridgeway Surgery, Harrow, London, United Kingdom

**Keywords:** antibiotics, dermatology, infectious diseases, practice management, venomous bites and stings

## Abstract

**Background:**

Insect bite inflammation may mimic cellulitis and promote unnecessary antibiotic usage, contributing to antimicrobial resistance in primary care. We wondered how general practice clinicians assess and manage insect bites, diagnose cellulitis, and prescribe antibiotics.

**Method:**

This is a Quality Improvement study in which 10 general practices in England and Wales investigated patients attending for the first time with insect bites between April and September 2021 to their practices. Mode of consultation, presentation, management plan, and reattendance or referral were noted. Total practice flucloxacillin prescribing was compared to that for insect bites.

**Results:**

A combined list size of 161,346 yielded 355 insect bite consultations. Nearly two-thirds were female, ages 3–89 years old, with July as the peak month and a mean weekly incidence of 8 per 100,000. GPs still undertook most consultations; most were phone consultations, with photo support for over half. Over 40% presented between days 1 and 3 and common symptoms were redness, itchness, pain, and heat. Vital sign recording was not common, and only 22% of patients were already taking an antihistamine despite 45% complaining of itch. Antibiotics were prescribed to nearly three-quarters of the patients, mainly orally and mostly as flucloxacillin. Reattendance occurred for 12% and referral to hospital for 2%. Flucloxacillin for insect bites contributed a mean of 5.1% of total practice flucloxacillin prescriptions, with a peak of 10.7% in July.

**Conclusions:**

Antibiotics are likely to be overused in our insect bite practice and patients could make more use of antihistamines for itch before consulting.

**Key messages**

- Insect bite inflammation and cellulitis are difficult to distinguish.
- Reattendance and referral are unusual.
- Oral antibiotic prescribing rates are high at first GP consultation.
- Flucloxacillin for insect bites contributed 5.1% of total prescribing (April–September).
- Itch is undertreated before consulting.

## Introduction

Evidence shows that general practice flucloxacillin prescribing increases in summer months, peaking in July.^1^ A specialist Insect Bite Group (IBG) within the Royal College of General Practitioners (RCGP) were considering whether this seasonal effect might be due to increased presentation with insect bites, and if so whether increased prescribing is appropriate or not. This IBG is within the RCGP overdiagnosis special interest group (OD). The OD group’s interest is in inappropriate medicalisation of self-limiting illness and over-use of antibiotics. The group includes general practitioners (GPs), including educators and researchers. We used Quality Improvement (QI) methodologies to undertake an initial analysis/assessment of this problem.

Best practice in the management of insect bites is not as clearly described as it is for other infective conditions such as tonsillitis.^2^ Current guidelines from the National Institute for Health and Care Excellence (NICE) on insect bites and stings^3^ state that “most will not require antibiotics” and recommends the use of antihistamines. GP clinicians (GPCs) may access allied guidance, for example, NICE guidance on impetigo^4^ recommends an antiseptic (hydrogen peroxide1% cream) for well patients with localised non-bullous impetigo, and, if not suitable, topical antibiotics. More widespread or severe non-bullous impetigo, and all bullous impetigo, are recommended a course of oral antibiotics, flucloxacillin, first line. Finally, NICE have guidance on erysipelas and cellulitis^5^ which recommends taking a swab from broken skin, drawing round the area with a pen, and offering an oral antibiotic, first-line flucloxacillin, it also recommends reassessing if worsening or not improving at 2 or 3 days. However, guidelines drawn from committee consensus and published evidence usually describes case studies of severe infection^6,7^ and may not translate to this common clinical scenario seen in primary care. Applying guidelines in practice requires key clinical judgements concerning which bites need medical management, or whether self-management is more appropriate. For GPCs, it can be difficult to distinguish clinical features of redness and heat from inflammatory reactions or secondary cellulitis.

To better understand current practice-based management of insect bites, the IBG opted to use a QI approach that aims to improve patient care through first understanding the problem in context, then using a systematic approach to examine, propose and implement change before assessing impact.^8^ Our study aimed to assess and understand the incidence of insect bite presentation, the management (and whether clinicians are following NICE guidelines^3^) and whether insect bite management contributes to the rise in summer flucloxacillin prescribing. We intend that our findings will inform practice improvement activity to address prescribing practice.

## Methods

### Research questions

The IBG identified the following research questions:

1. How commonly do insect bites present in general practices?
2. How do patients present to general practices, that is, what consultation modes are used?
3. What symptoms and signs of bites are recorded?
4. What treatment options are used?
5. How does insect bite-related flucloxacillin prescribing compare to practice-level flucloxacillin prescribing?

### Design

Our project adopted QI methods^8^ as these allow us to describe, critique, and potentially change current GPC practice. The first step in this approach is to define what we are trying to accomplish in order to generate ideas for change improvement. Our questions, therefore, focus on describing the size of the problem and the scope of current practice.

### Sampling frame

We explored the affected population and current service provision by GPCs in our IBG practices. This was a convenience sample of 10 practices, members of the RCGP OD group in England and Wales.

### Sample

Identifying all patients presenting with insect bites between the dates of 1st April to 30th September 2021, inclusive, by searching online all consultations using the words “insect bites” coded in participating practices. To identify consultations, inclusion criteria were: first consultation of any person, of any age, presenting with a presumed insect bite to general practices, even if they had contacted a different health care provider previously. Patients with insect bite reactions due to spiders or ticks (arthropods) were included if not Lyme disease, that is, managed as insect bites. It was accepted that it cannot be known whether some “bite” reactions might include vegetation injuries, for example, hogweed contact, but data were cleaned to exclude known stings and other causes.

Exclusion criteria: not first GPC consultations, stings, and illnesses GPCs diagnosed as not due to insect bites, that is, Lyme disease and a patient with shingles. Presentations to out-of-hours (OOH) or walk-in centres were excluded.

### Data extraction

For identified eligible consultations, the practice clinician extracted data related to the patient (age, sex), presenting problem (bite site, symptoms, duration), management plan (prescribing), follow-up or sequelae (re-presentation or referral to hospital), and service context (mode of consultation and clinician consulting). Data were collected using a study-specific instrument created by the team (see Appendix 1), drawing on evidence from our previous GP survey in this field,^9^ reading of the literature, and clinical expertise. This tool was applied by each of the investigators in their own practice, all data were anonymised and then exported to Microsoft Excel. Results were double checked by a second investigator.

### Analysis

Consultation data were converted into categorical (nominal data) where appropriate. The analysis used descriptive statistical analysis of practice-level clinical behaviour to answer research questions 1–4. An analysis plan was applied using descriptive statistics to assess incidence, demographics of patients, proportions of consultations by consultation modality and clinician, prevalence of symptoms/signs, and the proportions of treatment modalities. Results are to one decimal place. Question 5 used a different approach, based on analysis of the nine English practices, as data was not available for the Welsh tenth practice. This analysis sought to describe the proportion of flucloxacillin prescriptions issued at each practice accounted for by insect bite management. Data describing monthly prescribing of flucloxacillin at each of the 9 practices were identified from OpenPrescribing^10^ which is part of the Bennett Institute for applied data science at the University of Oxford and provides a search interface onto the raw English Prescribing Dataset published by NHS Business Services Authority and explores dispensed prescriptions. These data were compared to our observed flucloxacillin prescribed for insect bites in each practice.

The study, as QI, did not require NHS ethics or Health Research Authority approval, as it examines data on current provision of care. Information governance leads at each practice were consulted to check their agreement with the proposals. We use SQUIRE^11^ to report our findings. Two patient participation group members read either the initial data tool or draft paper to help improve the study report.

## Results

### Epidemiology (Table 1)

The combined practice list size was 161,346 people (range 8,196–53,232). Between 1st April and 30th September 2021, inclusive, there were 355 patients presenting as first attendance to their GPC with presumed insect bites in which GPCs also agreed the diagnosis. Of these, 64.8% were females and 35.2% were males. Ages ranged from 3 to 89 years old, with 51.5% of people being ages 30–59, with mode age decade being 50–59 years old (18%). June, July, and August accounted for 77.7% of bites in the study’s 6 months, and the most common month for insect bite presentations was July at 35.2% of the cohort. Only three bites occurred from abroad.

**Table 1.** Epidemiological data for sex, age decade and month of presentation for first attendance at general practice April–September inclusive 2021.

Criteria	Patient numbers	Percentage of 355 cohort bites (%)
Sex		
Male	125	35.2
Female	230	64.8
Age (3–89 years)		
0–9	17	4.8
10–19	30	8.5
20–29	47	13.2
30–39	62	17.5
40–49	57	16.1
50–59	64	18
60–69	46	13
70–79	27	7.6
80–89	5	1.4
Month		
April	18	5.1
May	12	3.4
June	74	20.9
July	125	35.2
August	77	21.7
September	49	13.8

A mean incidence of 0.2% of the population presented at least once to their GPC (range 0.1%–0.5%), that is 8 per 100,000 weekly, for the study 6 months.

### Consultation modalities (Table 2)

#### Consulting GPC:

GPs consulted in 59.7% of all consultations and 35.2% were with nurses.

**Table 2.** Consultation modality for insect bite first presentation to GPC April to September inclusive 2021.

Consultation personnel (GPc)	Numbers	% of insect bite cohort (355)
**GP total**	**212**	**59.7**
GP	182	51.2
GP locum	20	5.6
GP trainee	10	2.8
**Nurse total**	**125**	**35.2**
ANP	114	32.1
NPract.	7	2
PNurse	4	1.1
**Pharmacist**	**1**	**0.3**
**Unlisted**	**17**	**4.8**
Images used		
Yes	186	52.4
No	169	47.6
Modality		
**Phone total**	**248**	**69.9**
Phone alone	212	59.7
Phone and video	2	0.6
Phone converted to f2f	34	9.6
f2f alone	81	22.8
Video alone	4	1.1
**Digital consultation total**	**22**	**6.2**
Digital alone	11	3.1
Digital with photo	11	3.1

Note: Digital consultations are defined as remote with no voice input.

#### Consulting clinician before meeting the GPC

: 84.2% patients had not consulted another clinician. Community pharmacists had consulted with 4.2% patients, OOH clinicians with 2.3%, and a community optometrist with one patient.

#### Consultation modes

: Phone consultations occurred in 69.9% of the 355 consultations and 59.7% of the 355 cohort were as phone alone. Traditional face-to-face (f2f) alone consultations occurred in 22.8% of cases and another 9.6% of phone consultations were then converted to f2f. Total digital consultations (those with no voice or visual contribution), occurred in 3.1% of consults, and digital text with photo support in another 3.1% of cases. Video alone in 1.1% and video with phone consultation in 0.6%. Photographs were used to support 52.4% of the 355 consultations.

### Recorded features of bites (Table 3)

Time from the presentation was recorded in 79.2% and mode presentation was 1–3 days post bite, 42.5% of the cohort, with a range of presentations from within 1 day to over 3 weeks There were single bite presentations in 58.3% of cases and multiple bites in 38% with 3.7% unrecorded. The site was not recorded in 58% of cases, perhaps due to image use, but when recorded were mainly lower limb (27%) and 2.3% were recorded as facial, 0.9% involving the eye area.

**Table 3.** Recorded features of insect bites at first attendance to GPCs 355 April–September 2022 inclusive.

	Numbers of cases	Percentage (%) of 355 cohort
**Single bite**	207	58.3
**Multiple bite**	135	38
Unknown	13	3.7
**Length of time in days from insect bite to first GPc presentation**		
<1 day	15	4.2
1–3days	151	42.5
4–6 days	74	20.8
7–20 days	34	9.6
>21 days	7	2
Not recorded	74	20.8
**Anatomical sites (may be multiple)**		
Lower limb	96	27
Upper limb	36	10.1
Face (subgroup eye)	8 (3)	2.3
Abdo, back, neck	9	2.5
Not recorded	206	58
**Symptoms and sign**		
Itch	117	33
Itch and pain	44	12.4
Pain	68	19.2
Unknown itch/pain	126	35.5
Red	157	44.2
Red and heat	119	33.5
Heat	12	3.4
Unknown red and heat	67	18.9
Swelling	70	19.7
Discharge (subgroups pus, clear, unknown)	24 (plus 12, clear 1, unknown 11)	6.8
Symptoms of malaise	38	10.7
Pyrexia (>37.5C)	5	1.4
Vital signs	27	7.6

Recorded symptoms and signs were reported by patients or GPCs in the consultation. Redness in 77.7%, itch in 45.4%, heat in 36.9%, and pain occurred in 31.5%. Swelling was recorded in 19.7% and discharge in 6.8%. Systemic upset, for example, malaise, was recorded in 10.7% of cases, and 8.1% of malaise was reported in phone alone consultations. Pyrexia (>37.5C) was recorded in 1.4% and other vital signs, for example, pulse, in 7.6% of the cohort. We cannot report on comorbidities as it was unclear if these were used by GPCs accessing “digital problem screens” or not, nor could we analyse the size of bites due to uncertainty if size included areas of surrounding inflammation or not. No systemic allergic reactions presented, nor any other insect-borne diseases (Lyme disease cases had already been excluded).

### Medications and outcomes (Table 4)

#### Topical steroid use

Topical steroid use was advised or provided by the GPC in 15.2% of cases and 3.4% of patients were already using it. Oral steroid was prescribed for 2.5% of patients.

**Table 4.** Antibiotics used at first consultation for insect bites 1st April–30th September inclusive 2021.

Antibiotic at the first consultation with GPc	Numbers	Percentage of total cohort (355)%
Flucloxacillin	202	56.9
Clarithromycin	20	5.6
Doxycycline	10	2.8
Co-amoxiclav	9	2.5
Amoxicillin	3	0.8
Clindamycin	2	0.6
**Total oral antibiotics**	**246**	**69.3**
Topical fusidic acid	12	3.4
Topical mucipirin	1	0.3
Other (not listed if topical or oral)	6	1.7
**Total oral or topical antibiotics**	**265**	**74.7**
No antibiotic	86	24.2
No entry	4	1.1

#### Antihistamine (AH) use

: AH was not used in 47% of cases but was already being taken orally in 22%, and 2.5% topically before the first consultation with GPC. A GPC then advised or prescribed oral AH to 21.1% of patients and to five 1.4% topically.

#### Antibiotic prescriptions:

Oral and/or topical antibiotics were prescribed to 74.7% of the cohort as oral antibiotics to 69.3% and topical to 3.7%. Flucloxacillin made up 81.1% of the 246 oral antibiotic prescriptions and was prescribed to 56.9% of the cohort. No antibiotic on first attendance to a GPC was given in 24.2% of cases.

### Reattendance

Reattendance to any clinician occurred for 12.1% of the cohort, and 2% were referred to hospitals or outpatients for further care. Data on referral were recorded for four patients, two as osteomyelitis, one thrombophlebitis, and one admitted with “infection”.

### Total flucloxacillin prescribing compared to that for insect bites

**Figure 1.**
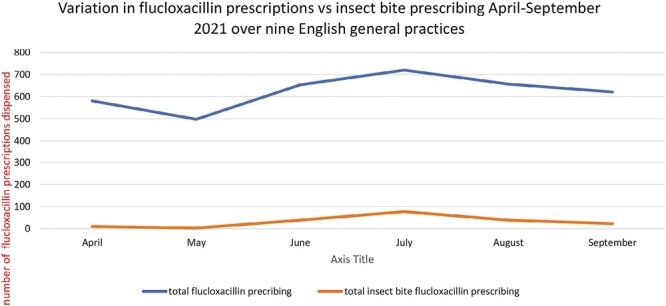
Graph of flucloxacillin prescribing.

For the nine English general practices, data on total flucloxacillin dispensing was available monthly for each practice from April to September using OpenPrescribing^10^ and so could be compared relative to insect bite flucloxacillin prescribing from the investigating practices (Fig. 1). There were 3731 flucloxacillin prescriptions prescribed and dispensed and from this study, we know 191 were by first GPC consultations for insect bites. Therefore, a mean of 5.1% of all flucloxacillin prescriptions was for the first GPC insect bite treatment over the 6 months, minimum of 0.6% in May to 10.7% in July. During the peak of July, there were 720 flucloxacillin prescriptions dispensed and 77 prescribed for insect bite management. If these 77 prescriptions were all dispensed, then 10.7% of flucloxacillin prescriptions were for insect bite management in July 2021.

## Conclusion

These results highlight a mean incidence of first presentation to general practices of insect bites of 8 per 100,000 population weekly over the 6 months of the study. A study^12^ of 1999–2003 exploring both insect bites and impetigo reported an incidence of 5.4 per 100,000 population of England and Wales over 12 months and as insect bite incidence reduces in the winter our figure is comparable but does not represent additional consultations by alternative primary care providers.

In contrast to NICE guidance,^3^ 74% of our patients received an oral or topic antibiotic, most commonly flucloxacillin. This choice adheres to guideline recommendations for cellulitis^5^ and some prescribing reflected reported patient adverse reactions or allergies. Flucloxacillin for insect bites is, therefore, a small contributor to the rises in flucloxacillin practice prescribing over the summer months. We found a difference between the previously reported idealisation of practice by surveyed GPs^9^ and practice-based clinician activity, for example, vital signs were not prime factors in antibiotic prescribing. Either there is a high incidence of cellulitis or there is overprescribing, with scope to improve antibiotic stewardship by exploring management as the next stage of QI. Although a small effect at the level of ten practices, the potential impact on antibiotic stewardship at the UK level is significant.

In addition, most patients still contact their general practices first but meet an expanded clinical team with nurses over a third of consulters. Almost 70% of consultations start by phone and less than 10% are converted to f2f consultations. Images support consultations in just over half of interactions. Most people present 1–7 days post bite, most commonly days 1–3 with common features of redness, itchness, pain, and swelling, July was the peak month. A review of cellulitis^13^ gave key symptoms as pain, swelling, and heat, similar to our study, except we also had itching as a key symptom. Only 22% of patients used oral antihistamine before consultation, despite 45.4% complaining of itch. The use of antihistamines, as recommended by NICE,^3^ may be an area for improvement, although there is uncertainty about their efficacy in reducing itch or cellulitis complications and this is a priority area for future research. Topical steroids were not commonly used and when used were mainly hydrocortisone cream. There is a need to establish the efficacy and best strength of local steroid to reduce inflammation, and itch and prevent secondary cellulitis.

Our novel approach seeks to generate real-world practice-based evidence using QI approaches. The IBG is a professionally led group based within the RCGP using practice-based research to exchange knowledge with colleagues, analyse patient health care use, explore management dilemmas, and define future research activity. This is the first reporting on management of a cohort of primary care insect bite practice to our knowledge. QI approaches create some limitations as insect bites were selected by searching on coded data, some may therefore be missing from the data set. The number of total bites occurring in the population and self-managed, versus the number presenting to all primary care providers versus those presenting to general practices is unknown. Patients may also attend walk-in clinics, out of hours services, community pharmacists, and A&E departments for insect bite concerns. Our practices were not randomised and practice list size varied greatly, introducing possible bias. During the COVID-19 pandemic, a study^14^ reported an overall unchanged prescribing of antibiotics compared to that forecast, but a reduction of flucloxacillin prescribing of 12.7% compared to forecasts for the month of July 2020. We do not know how this may have affected our study. Reduced social contact, and reduced travel abroad but increased exercise in local open spaces will have created some changes to patients’ behaviour and also to pandemic prescribing. Our study reflects insect bite issues from England and Wales, rather than travel abroad, which was much more common before the COVID-19 pandemic years. We cannot draw any conclusions about overseas bites, and this is an area for future study. We have assumed that antibiotic prescriptions are prescribed and taken by patients, this may not be the case as some were asked to use as a delayed course, in case of deterioration.

Our data suggests there could be scope to change and improve the management of insect bites, with greater use of non-antibiotic management. To help detect early cellulitis swab of purulent discharge and drawing round the erythema to monitor spread may be helpful. Exploration of antihistamine, topical steroid, and topical antiseptics in insect bite management may reduce antibiotic use. Our findings highlight the next steps needed in a QI approach to generate practice-based evidence on optimal management of insect bites, specifically work on alternatives to antibiotic prescribing.

The James Lind alliance^15^ has worked with patients to identify research questions related to the management of cellulitis, including “What are the early signs and symptoms of cellulitis that can help to ensure speedy treatment?”. For insect bites in general practice, we suggest “What early symptoms and signs in insect bite reactions do not require antibiotics?” and “What non-antibiotic treatments reduce inflammation and cellulitis?” as changes to design and implement as the next step in a QI model to develop practice-based evidence.

## Supplementary Material

cmad051_suppl_Supplementary_Appendix_1

cmad051_suppl_Supplementary_Checklist

## Data Availability

Data available on request to the corresponding author for 5 years.
