# Supplementary material for: Are insect bites responsible for the rise in summer flucloxacillin prescribing in United Kingdom general practices?
Source: Fam Pract. 2023 May 6;40(5-6):753–9. doi: 10.1093/fampra/cmad051 (PMC10745258; doi:10.1093/fampra/cmad051)
Supplement: cmad051_suppl_Supplementary_Appendix_1 [file cmad051_suppl_supplementary_appendix_1.docx]

**Appendix 1 Data collection**

Each practice determines:

Drop down menus and numbers. Unknown was included in options. Two areas of free text in ‘comorbidity’ and ‘reason for presentation’ included

- Person identification number e.g., 1-100
- Age in years, report a zero for a baby up to 1 years old. Later categories to decades: 1-9, 10-19 etc.
- Sex assigned at time of consultation: male, female
- Relevant comorbidity considered by assessing clinician e.g., immunosuppression, diabetes, eczema. Free Text
- Month of first consultation to general practice clinician (GPc)
- First GPc consulted i.e., GP, GP locum, GP trainee, ANP, PA, NP, PN, student nurse, pharmacist, other
- Mode of consultation: total digital, digital text with photo, phone, video, phone & video, phone & then f2f, face to face (f2f)
- Photo images used yes, no
- Bite geographical location: unknown, United Kingdom, abroad
- Referred from another clinician: none, unknown, pharmacy, nurse, OOH, other
- Reason for consult including any progression of symptoms or/and speed of progression including site of bite if recorded. Free Text
- Number of bites: unknown, single, multiple
- Size of bite diameter: 1-5mm, 5-10mm, 1-5cm, 5-10cm and over 10cm
- Days since bite onset (from first presentation if multiple attendances): unknown, within one day, 1-3 days 4-7 days, 1-3 weeks, >3 weeks
- Local inflammation signs: unknown, red, red & heat, heat
- Spreading inflammation: unknown, regional inflammation, lymphangitis, regional lymph nodes.
- Bite appearance: raised (wheal), flat, blister, other, unknown
- Itch and Pain present: none, itch, itch& pain, pain, unknown
- Systemic upset recorded e.g., malaise, sweating: yes, no
- Temperature recorded and if pyrexia (value>37.5C): none, pyrexia, unknown
- Other systemic signs recorded e.g., BP, pulse, SpO2: yes, no
- Home remedies e.g., analgesia or cooling started by patient (not antihistamine and not steroids): unknown, cooling, paracetamol, NSAID, NSAID & paracetamol, herbal, other
- Antihistamine use, by patient or clinician: none, already using oral, already using topical, advised or prescribed oral, advised or prescribed topical
- Topical steroid prescribed or advised: none, already using, advised or prescribed topical, oral steroid
- Antibiotic prescription: none, flucloxacillin, clarithromycin, erythromycin, phenoxymethylpenicillin, co-amoxiclav, doxycycline, amoxicillin, other (later at analysis other was relisted as other, topical fusidic acid, topical mupirocin, oral clindamycin)
- Refer/admit: no referral, asked for phone advice, referred to OPC, sent to hospital, unknown
- Reattendance: unknown, yes once, yes more than once, none
- Comments. Free text

Abbreviations: f2f face to face; GP general practitioner; ANP advance nurse practitioner; PA physician associate; NP nurse practitioner; PN practice nurse; OOH out-of -hours primary care service; BP blood pressure; SpO2 oximetry finger oxygen level; NSAID non- steroidal anti-inflammatory drugs; OPC out- patient clinic.
