# Supplementary material for: Are insect bites responsible for the rise in summer flucloxacillin prescribing in United Kingdom general practices?
Source: Fam Pract. 2023 May 6;40(5-6):753–9. doi: 10.1093/fampra/cmad051 (PMC10745258; doi:10.1093/fampra/cmad051)
Supplement: cmad051_suppl_Supplementary_Checklist [file cmad051_suppl_supplementary_checklist.docx]

# P2Reporting checklist for quality improvement in health care.

Based on the SQUIRE guidelines.

## Instructions to authors

Complete this checklist by entering the page numbers from your manuscript where readers will find each of the items listed below.

Your article may not currently address all the items on the checklist. Please modify your text to include the missing information. If you are certain that an item does not apply, please write "n/a" and provide a short explanation.

Upload your completed checklist as an extra file when you submit to a journal.

In your methods section, say that you used the SQUIRE reporting guidelines, and cite them as:

Ogrinc G, Davies L, Goodman D, Batalden P, Davidoff F, Stevens D. SQUIRE 2.0 (Standards for QUality Improvement Reporting Excellence): revised publication guidelines from a detailed consensus process

|  |  | Reporting Item | Page Number |
| --- | --- | --- | --- |
| **Title** |  |  |  |
|  | Title page P1 | Indicate that the manuscript concerns an initiative to improve healthcare (broadly defined to include the quality, safety, effectiveness, patientcenteredness, timeliness, cost, efficiency, and equity of healthcare)  **Are insect bites responsible for the rise in summer flucloxacillin prescribing in UK general practices?** |  |
| **Abstract** |  |  |  |
|  | Abstract P2 | Provide adequate information to aid in searching and indexing |  |
|  |  | Summarize all key information from various sections of the text using the abstract format of the intended publication or a structured summary such as: background, local problem, methods, interventions, results, conclusions  **Background.** Insect bite inflammation may mimic cellulitis and promote unnecessary antibiotic usage, contributing to antimicrobial resistance in primary care. We wondered how general practice clinicians assess and manage insect bites, diagnose cellulitis and prescribe antibiotics.  **Method.** This is a Quality Improvement study in which ten general practices in England and Wales investigated patients attending for the first time with insect bites between April and September 2021 to their practices. Mode of consultation, presentation, management plan and reattendance or referral were noted. Total practice flucloxacillin prescribing was compared to that for insect bites.  **Results.** A combined list size of 161,346 yielded 355 insect bite consultations. Nearly two thirds were female, ages 3-89 years old, with July as the peak month and a mean weekly incidence of 8 per 100,000. GPs still undertook most consultations; most were phone consultations, with photo support for over half. Over 40% presented between days 1-3 and common symptoms were redness, itch, pain, heat. Vital sign recording was not common and only 22% of patients were already taking an antihistamine despite 45% complaining of itch. Antibiotics were prescribed to nearly three quarters of the patients, mainly orally and mostly as flucloxacillin. Reattendance occurred for 12% and referral to hospital for 2%. Flucloxacillin for insect bites contributed a mean of 5.1% of total practice flucloxacillin prescriptions, with a peak of 10.7% in July.  **Conclusions.** Antibiotics are likely to be overused in our insect bite practice and patients could make more use of antihistamines for itch before consulting. |  |
| **Introduction** |  |  |  |
| Problem description | P4 | Nature and significance of the local problem  Evidence shows that general practice flucloxacillin prescribing increases in summer months, peaking in July^1^. A specialist Insect Bite Group (IBG) within the Royal College of General Practitioners (RCGP) were considering whether this seasonal effect might be due to increased presentation with insect bites, and if so whether increased prescribing is appropriate or not. |  |
| Available knowledge | P2 | Summary of what is currently known about the problem, including relevant previous studies  Best practice in management of insect bites is not as clearly described as it is for other infective conditions such as tonsillitis^2^. Current guidelines from the National Institute for Health and Care Excellence (NICE) on insect bites and stings^3^ states that ‘most will not require antibiotics’ and recommends use of antihistamines. GP clinicians (GPCs) may access allied guidance e.g. NICE guidance on impetigo^4^ recommends an antiseptic (hydrogen peroxide1% cream) for well patients with localised non-bullous impetigo, and, if not suitable, topical antibiotics. More widespread or severe non- bullous impetigo, and all bullous impetigo, are recommended a course of oral antibiotics, flucloxacillin, first line. Lastly, NICE have guidance on erysipelas and cellulitis^5^ which recommends taking a swab from broken skin, drawing round the area with a pen and offering an oral antibiotic, first line flucloxacillin, it also recommends reassessing if worsening or not improving at 2 or 3 days. |  |
| Rationale | P5 | Informal or formal frameworks, models, concepts, and / or theories used to explain the problem, any reasons or assumptions that were used to develop the intervention(s), and reasons why the intervention(s) was expected to work  To better understand current practice -based management of insect bites the IBG opted to use a QI approach which aims to improve patient care through first understanding the problem in context, then using a systematic approach to examine, propose and implement change, before assessing impact^8^. |  |
| Specific aims | P[5](https://www.goodreports.org/reporting-checklists/squire/info/#6) | Purpose of the project and of this report |  |
| **Methods** |  | Our study aimed to assess and understand the incidence of insect bite presentation, the management (and whether clinicians are following NICE guidelines^3^) and whether insect bite management contributes to the rise in summer flucloxacillin prescribing. We intend that our findings will inform practice improvement activity to address prescribing practice. |  |
| Context | P5 | Contextual elements considered important at the outset of introducing the intervention(s)  *Design:* Our project adopted QI methods as these allow us to describe, critique and potentially change current GPC practice. The first step in this approach is to define what we are trying to accomplish in order to generate ideas for change improvement. Our questions therefore focus on describing the size of the problem and scope of current practice.  *Sampling frame:* We explored the affected population and current service provision by GPCs in our IBG practices. This was a convenience sample of 10 practices, members of the RCGP OD group in England and Wales. |  |
| Intervention(s) | PP5-6 | Description of the intervention(s) in sufficient detail that others could reproduce it  cohort case study and analysis  *Sample:* Identifying all patients presenting with insect bites between dates of 1^st^ April to 30^th^ September 2021 inclusive, by searching online all consultations using words ‘insect bites’ coded in participating practices. To identify consultations, inclusion criteria were: first consultation of any person, of any age, presenting with a presumed insect bite to general practices, even if they had contacted a different health care provider previously. Patients with insect bite reactions due to spiders or ticks (arthropods), were included if not Lyme disease i.e. managed as insect bites. It was accepted that it cannot be known whether some ‘bite’ reactions might include vegetation injuries e.g. hogweed contact, but data was cleaned to exclude known stings and other causes.  Exclusion criteria were: not first GPC consultations, stings and illnesses GPCs diagnosed as not insect bites i.e. Lyme disease and a patient with shingles. Presentations to out-of-hours (OOH) or walk in centres (WIC) were excluded. |  |
| Intervention(s) | P5  and P4 | Specifics of the team involved in the work  *Sampling frame:* We explored the affected population and current service provision by GPCs in our IBG practices. This was a convenience sample of 10 practices, members of the RCGP OD group in England and Wales.  A specialist Insect Bite Group (IBG) within the Royal College of General Practitioners (RCGP) were considering whether this seasonal effect might be due to increased presentation with insect bites, and if so whether increased prescribing is appropriate or not. This IBG is within the RCGP overdiagnosis special interest group (OD). The OD group’s interest is in inappropriate medicalisation of self- limiting illness and over-use of antibiotics. The group includes general practitioners (GPs), including educators and researchers. We used Quality Improvement (QI) methodologies to undertake an initial analysis/assessment of this problem. |  |
| Study of the Intervention(s) | NA | Approach chosen for assessing the impact of the intervention(s)  There is no change intervention as the management of insect bites is not yet reported in primary care so we are producing data for step 1 of QI, defining the issue and potential problems |  |
| Study of the Intervention(s) | NA | Approach used to establish whether the observed outcomes were due to the intervention(s) |  |
| Measures | P6 | Measures chosen for studying processes and outcomes of the intervention(s), including rationale for choosing them, their operational definitions, and their validity and reliability  *Data extraction:* For identified eligible consultations, the practice clinician extracted data related to the patient (age sex), presenting problem (bite site, symptoms, duration), management plan (prescribing), follow up or sequelae (re-presentation or referral to hospital) and service context (mode of consultation and clinician consulting). Data was collected using a study specific instrument created by the team (see appendix1), drawing on evidence from our previous GP survey in this field^9^, reading of the literature and clinical expertise. This tool was applied by each of the investigators in their own practice, all data was anonymised and then exported to Microsoft Excel. |  |
| Measures | NA | Description of the approach to the ongoing assessment of contextual elements that contributed to the success, failure, efficiency, and cost  There is not an intervention or cost |  |
| Measures | P6 | Methods employed for assessing completeness and accuracy of data  This tool was applied by each of the investigators in their own practice, all data was anonymised and then exported to Microsoft Excel. Results were double checked by a second investigator. |  |
| Analysis | P6 | Qualitative and quantitative methods used to draw inferences from the data  Consultation data was converted into categorical (nominal data) where appropriate. The analysis used descriptive statistical analysis of practice level clinical behaviour to answer research questions 1-4. An analysis plan was applied using descriptive statistics to assess incidence, demographics of patients, proportions of consultations by consultation modality and clinician, prevalence of symptoms/signs and the proportions of treatment modalities. Results are to one decimal place. Question 5 used a different approach, based on analysis of the nine English practices, as data was not available for the Welsh tenth practice. This analysis sought to describe the proportion of flucloxacillin prescriptions issued at each practice accounted for by insect bite management. Data describing monthly prescribing of flucloxacillin at each of the 9 practices was identified from OpenPrescribing^10^ which is part of the Bennett Institute for applied data science at the University of Oxford and provides a search interface onto the raw [English Prescribing Dataset](about:blank) published by NHS Business Services Authority and explores dispensed prescriptions. This data was compared to our observed flucloxacillin prescribed for insect bites in each practice. |  |
| Analysis | P11 | Methods for understanding variation within the data, including the effects of time as a variable  We have no intervention, the base line data of insect bite practice is reported, possible variation is discussed as limitations on P11 and pasted below, and in analysis above we discuss how we didnt include the Welsh practice in total flucloxacillin prescribing due to variations in prescribing and dispensing recording  QI approaches create some limitations as insect bites were selected by searching on coded data, some may therefore be missing from the data set. The numbers of total bites occurring in the population and self- managed, versus the number presenting to all primary care providers versus those presenting to general practices is unknown. Patients may also attend WICs, OOHs, community pharmacists, A&E departments for insect bite concerns. Our practices were not randomised and practice list size varied greatly, introducing possible bias. During the COVID-19 pandemic a study^14^ reported an overall unchanged prescribing of antibiotics compared to that forecast, but a reduction of flucloxacillin prescribing of 12.7% compared to forecasts for the month of July 2020. We do not know how this may have affected our study. Reduced social contact, reduced travel abroad but increased exercise in local open spaces will have created some changes to patients’ behaviour and also to pandemic prescribing. Our study reflects insect bite issues from England and Wales, rather than travel abroad, than in other years. We can’t draw any conclusions about overseas bites, and this is an area for future study. We have assumed that antibiotic prescriptions are prescribed and taken by patients, this may not be the case as some were asked to use as a delayed course, in case of deterioration. |  |
| Ethical considerations | P7 | Ethical aspects of implementing and studying the intervention(s) and how they were addressed, including, but not limited to, formal ethics review and potential conflict(s) of interest  The study, as QI, did not require NHS ethics or Health Research Authority approval, as it examines data on current provision of care. Information governance leads at each practice were consulted, to check their agreement with the proposals  This study is not funded and there are no conflicts of interest |  |
| **Results** |  |  |  |
|  | NA | Initial steps of the intervention(s) and their evolution over time (e.g., time-line diagram, flow chart, or table), including modifications made to the intervention during the project  There is no intervention |  |
|  | P6 | Details of the process measures and outcome  Consultation data was converted into categorical (nominal data) where appropriate. The analysis used descriptive statistical analysis of practice level clinical behaviour to answer research questions 1-4. |  |
|  | NA | Contextual elements that interacted with the intervention(s)  No intervention |  |
|  | P7 | Observed associations between outcomes, interventions, and relevant contextual elements  *Epidemiology (table 1):* The combined practice list size was 161,346 people (range 8,196 - 53,232). Between 1^st^ April - 30^th^ Sept 2021 inclusive there were 355 patients presenting as first attendance to their GPC with presumed insect bites in which GPCs also agreed the diagnosis. Of these, 230 (64.8%) were females and 125 (35.2%) males. Ages ranged from 3-89 years old, with 51.5% of people being ages 30 – 59, with mode age decade being 50-59 years old (18%). June, July and August accounted for 77.7% of bites in the study six months, and the most common month for insect bite presentations was July at 35.2% of the cohort. Only three bites occurred from abroad.  A mean incidence of 0.2% of the population presented at least once to their GPC (range 0.1% - 0.5%), that is 8 per 100,000 weekly for the study 6 months.  *Consultation modalities (table 2):*  *Consulting GPC;* GPs consulted in 59.7% of all consultations and 35.2% were with nurses.  *Consulting clinician before meeting the GPC;* 299 (84.2%) patients had not consulted another clinician. Community pharmacists had consulted with 15 (4.2%) patients, OOH clinicians eight (2.3%) and a community optometrist one patient.  *Consultation modes;* Phone consultations occurred in 69.9% of the 355 consultations and 59.7% of the 355 cohort were as phone alone. Traditional face-to-face (f2f) alone consultations occurred in 22.8% of cases and another 34 phone consultations then converted to f2f (9.6%). Total digital consultations, (those with no voice or visual contribution), occurred in 3.1% of consults and digital text with photo support in another 3.1% of cases. Video alone in 1.1% and video with phone consultation in 0.6%. Photographs were used to support 186 of the 355 consultations (52.4%).  *Recorded features of bites (table 3):*  Time from presentation was recorded in 281 (79.2%) and mode presentation was from 1-3 days post bite (151 or 42.5%) of the cohort, with range of presentations from within one day to over 3 weeks There were single bite presentations in 207 (58.3%) cases and multiple bites in 135 (38%) with 13 (3.7%) unrecorded. Site was not recorded in 58% of cases, perhaps due to image use, but when recorded were mainly lower limb (27%) and 2.3% were recorded as facial, 0.9% involving the eye area.  Recorded symptoms and signs were reported by patients or GPCs in the consultation. Pain occurred in 31.5%, itch in 45.4%, redness in 77.7% and heat in 36.9%. Swelling was recorded in 19.7% and discharge in 6.8%. Systemic upset e.g. malaise, was recorded in 10.7% of cases and 8.1% of malaise was reported in phone alone consultations. Pyrexia (>37.5C) was recorded in 1.4% and other vital signs, e.g. pulse, in 7.6% of the cohort. We cannot report on comorbidities as it was unclear if these were used by GPCs accessing ‘digital problem screens’ or not, nor could we analyse size of bites, due to uncertainty if size included areas of surrounding inflammation or not. No systemic allergic reactions presented, nor any other insect borne diseases (Lyme disease cases had already been excluded).  *Medications and outcomes (table 4):*  *Topical steroid use;* was advised or provided by the GPC in 54 (15.2%) of cases and 12 (3.4%) of patients were already using it. Oral steroid was prescribed for nine (2.5%) patients.  *Antihistamine (AH) use*; AH was not used in 168 (47%) of cases but was already being taken orally in 78 (22%), and nine (2.5%) topically before first consultation with GPC. A GPC then advised or prescribed oral AH to 75 (21.1%) patients and to five (1.4%) topically.  *Antibiotic prescriptions;* Oral and/or topical antibiotics were prescribed to 265 (74.7%) of the cohort as oral antibiotics 246 (69.3%) and topical 13 (3.7%). Flucloxacillin made up 202 (81.1%) of the 246 oral antibiotic prescriptions and was prescribed to 56.9% of the cohort. No antibiotic on first attendance to a GPC was given in 86 (24.2%) cases.  *Reattendance*  Reattendance to any clinician occurred for 12.1% of the cohort. Seven patients (2%) were referred to hospital or outpatients for further care. Data on referral was recorded for four patients, two as osteomyelitis, one thrombophlebitis and one admitted with infection.  *Total flucloxacillin prescribing compared to that for insect bites*  For the nine English general practices, data on total flucloxacillin dispensing was available monthly for each practice from April to September using OpenPrescribing^10^and so could be compared relative to insect bite flucloxacillin prescribing from the investigating practices (figure 1). There were 3,731 flucloxacillin prescriptions prescribed and dispensed and from this study we know 191 were by first GPC consultations for insect bites. Therefore, a mean of 5.1% of all flucloxacillin prescriptions were for first GPC insect bite treatment over the six months, minimum 0.6% in May to 10.7% in July. During the peak of July there were 720 flucloxacillin prescriptions dispensed and 77 prescribed for insect bite management. If these 77 prescriptions were all dispensed, then 10.7% of flucloxacillin prescriptions were for insect bite management in July 2021. |  |
|  | P11 | Unintended consequences such as unexpected benefits, problems, failures, or costs associated with the intervention(s).  See above on study limitations, copied below:  QI approaches create some limitations as insect bites were selected by searching on coded data, some may therefore be missing from the data set. The numbers of total bites occurring in the population and self- managed, versus the number presenting to all primary care providers versus those presenting to general practices is unknown. Patients may also attend WICs, OOHs, community pharmacists, A&E departments for insect bite concerns. Our practices were not randomised and practice list size varied greatly, introducing possible bias. During the COVID-19 pandemic a study^14^ reported an overall unchanged prescribing of antibiotics compared to that forecast, but a reduction of flucloxacillin prescribing of 12.7% compared to forecasts for the month of July 2020. We do not know how this may have affected our study. Reduced social contact, reduced travel abroad but increased exercise in local open spaces will have created some changes to patients’ behaviour and also to pandemic prescribing. Our study reflects insect bite issues from England and Wales, rather than travel abroad, than in other years. We can’t draw any conclusions about overseas bites, and this is an area for future study. We have assumed that antibiotic prescriptions are prescribed and taken by patients, this may not be the case as some were asked to use as a delayed course, in case of deterioration. |  |
|  | P11 | Details about missing data  QI approaches create some limitations as insect bites were selected by searching on coded data, some may therefore be missing from the data set. T |  |
| **Discussion** |  |  |  |
| Summary | P5 | Key findings, including relevance to the rationale and specific aims   1. The specific research questions are 2. How commonly do insect bites present to general practices? 3. How do patients present to general practices, i.e. what consultation modes are used? 4. What symptoms and signs of bites are recorded? 5. What treatment options are used? 6. Is insect bite related flucloxacillin prescribing associated with practice level flucloxacillin prescribing? |  |
| Summary | P10-11 | Particular strengths of the project  We found a difference between previously reported idealisation of practice by surveyed GPs^9^ and practice-based clinician activity e.g. vital signs were not prime factors in antibiotic prescribing  and  Our novel approach seeks to generate real-world practice- based evidence using QI approaches. The IBG is a professionally led group based within the RCGP using practice-based research to exchange knowledge with colleagues, analyse patient health care use, explore management dilemmas and define future research activity. This is the first reporting on management of a cohort of primary care insect bite practice to our knowledge |  |
| Interpretation | P10 | Nature of the association between the intervention(s) and the outcomes  No intervention but the nature of the outcomes is here:  These results highlight a mean incidence of first presentation to general practices of insect bites of 8 per 100,000 population weekly over the six months of the study. A study^12^ of 1999-2003 exploring both insect bites and impetigo reported an incidence of 5.4 per 100,000 population of England and Wales over 12 months and as insect bite incidence reduces in the winter our figure is comparable but does not represent additional consultations by alternative primary care providers.  In contrast to NICE guidance^3^, 74% of our patients received an oral or topic antibiotic, most commonly flucloxacillin. This choice adheres to guideline recommendations for cellulitis^5^ and some prescribing reflected reported patient adverse reactions or allergies. Flucloxacillin for insect bites is therefore a small contributor to rises in flucloxacillin practice prescribing over the summer months. We found a difference between previously reported idealisation of practice by surveyed GPs^9^ and practice-based clinician activity e.g. vital signs were not prime factors in antibiotic prescribing. Either there is a high incidence of cellulitis or there is overprescribing, with scope to improve antibiotic stewardship by exploring management as the next stage of QI. Although a small effect at the level of ten practices, the potential impact on antibiotic stewardship at UK level is significant.  In addition, most patients still contact their general practices first but meet an expanded clinical team with nurses over a third of consulters. Almost 70% of consultations start by phone and less than 10% are converted to f2f consultations. Images support consultations in just over half of interactions. Most people present 1-7 days post bite, most commonly days 1-3 with common features of redness, itch, pain and swelling, July was the peak month. A review of cellulitis^13^ gave key symptoms as pain, swelling and heat, similar to our study, except we also had itch as a key symptom. As only 22% of patients used oral antihistamine before consultation, despite 45.4% complaining of itch, yet it is recommended by NICE^3^ this may be an area for improvement. |  |
| Interpretation | P10  P11 | Comparison of results with findings from other publications  These results highlight a mean incidence of first presentation to general practices of insect bites of 8 per 100,000 population weekly over the six months of the study. A study^12^ of 1999-2003 exploring both insect bites and impetigo reported an incidence of 5.4 per 100,000 population of England and Wales over 12 months and as insect bite incidence reduces in the winter our figure is comparable but does not represent additional consultations by alternative primary care providers.  and  In contrast to NICE guidance^3^, 74% of our patients received an oral or topic antibiotic, most commonly flucloxacillin. This choice adheres to guideline recommendations for cellulitis^5^ and some prescribing reflected reported patient adverse reactions or allergies. Flucloxacillin for insect bites is therefore a small contributor to rises in flucloxacillin practice prescribing over the summer months. We found a difference between previously reported idealisation of practice by surveyed GPs^9^ and practice-based clinician activity e.g. vital signs were not prime factors in antibiotic prescribing  and  During the COVID-19 pandemic a study^14^ reported an overall unchanged prescribing of antibiotics compared to that forecast, but a reduction of flucloxacillin prescribing of 12.7% compared to forecasts for the month of July 2020. We do not know how this may have affected our study. |  |
| Interpretation | P10 | Impact of the project on people and systems  Either there is a high incidence of cellulitis or there is overprescribing, with scope to improve antibiotic stewardship by exploring management as the next stage of QI. Although a small effect at the level of ten practices, the potential impact on antibiotic stewardship at UK level is significant. |  |
| Interpretation | NA | Reasons for any differences between observed and anticipated outcomes, including the influence of context  This study sets the baseline data for general practice insect bite management, no intervention |  |
| Interpretation | NA | Costs and strategic trade-offs, including opportunity costs |  |
| Limitations | P10 | Limits to the generalizability of the work  As above  Patients may also attend WICs, OOHs, community pharmacists, A&E departments for insect bite concerns. Our practices were not randomised and practice list size varied greatly, introducing possible bias. During the COVID-19 pandemic a study^14^ reported an overall unchanged prescribing of antibiotics compared to that forecast, but a reduction of flucloxacillin prescribing of 12.7% compared to forecasts for the month of July 2020. We do not know how this may have affected our study. Reduced social contact, reduced travel abroad but increased exercise in local open spaces will have created some changes to patients’ behaviour and also to pandemic prescribing. Our study reflects insect bite issues from England and Wales, rather than travel abroad, than in other years. We can’t draw any conclusions about overseas bites, and this is an area for future study. We have assumed that antibiotic prescriptions are prescribed and taken by patients, this may not be the case as some were asked to use as a delayed course, in case of deterioration. |  |
| Limitations | P10 | Factors that might have limited internal validity such as confounding, bias, or imprecision in the design, methods, measurement, or analysis  As above |  |
| Limitations | NA | Efforts made to minimize and adjust for limitations |  |
| Conclusion | P11 | Usefulness of the work  Our data suggests there could be scope to change and improve management of insect bites, with greater use of non-antibiotic management |  |
| Conclusion | NA | Sustainability |  |
| Conclusion | NA | Potential for spread to other contexts |  |
| Conclusion | P11 | Implications for practice and for further study in the field  A review of cellulitis gave key symptoms as pain, swelling and heat, similar to our study, except we also had itch as a key symptom. As only 22% of patients used oral antihistamine before consultation, despite 45.4% complaining of itch, yet it is recommended by NICE^3^ this may be an area for improvement.  Our findings highlight the next steps needed in a QI approach to generate practice-based evidence on optimal management of insect bites, specifically work on alternatives to antibiotic prescribing |  |
| Conclusion | P11 | Suggested next steps  The James Lind alliance^15^ have worked with patients to identify research questions related to the management of cellulitis, including ‘What are the early signs and symptoms of cellulitis that can help to ensure speedy treatment?’. For insect bites in general practice, we suggest ‘What early symptoms and signs in insect bite reactions do not require antibiotics?’ and ‘What non-antibiotic treatments reduce inflammation and cellulitis?’ as changes to design and implement as the next step in a QI model to develop practice-based evidence. |  |
| **Other information** |  |  |  |
| Funding | NA | Sources of funding that supported this work. Role, if any, of the funding organization in the design, implementation, interpretation, and reporting  No funding |  |

None The SQUIRE 2.0 checklist is distributed under the terms of the Creative Commons Attribution License CC BY-NC 4.0. This checklist can be completed online using <https://www.goodreports.org/>, a tool made by the [EQUATOR Network](https://www.equator-network.org) in collaboration with [Penelope.ai](https://www.penelope.ai)
